# A Multidisciplinary Approach in the Management of Aggressive Retinopathy of Prematurity With Bleb-Like Posterior Combined Retinal Detachment

**DOI:** 10.7759/cureus.103144

**Published:** 2026-02-07

**Authors:** K Shreeya Jain, Akash Belenje

**Affiliations:** 1 Vitreoretina, Srimati Kanuri Santhamma Center for Vitreo Retinal Diseases, Anant Bajaj Retina Institute, Standard Chartered-LVPEI Academy for Eye Care Education, L V Prasad Eye Institute, Hyderabad, IND

**Keywords:** aggressive retinopathy of prematurity, anaemia correction, bleb-like posterior combined retinal detachment, lens sparing vitrectomy, role of multidisciplinary approach

## Abstract

We report two cases of bleb-like combined retinal detachment (BLRD) in infants with aggressive retinopathy of prematurity (A-ROP). Both cases involved premature infants born at 28 weeks of gestation with complex postnatal courses, who were referred to our clinic for ROP management. On presentation, both babies were diagnosed with stage 4B ROP in zone 1 with bilateral BLRD. Indirect ophthalmoscope-guided laser photocoagulation under topical anesthesia was performed on the same day. Pediatric clearance for surgery was obtained. Both infants underwent blood transfusions for anemia. Subsequently, 25-gauge lens-sparing vitrectomy with endolaser and fluid-air exchange was performed in both cases. Postoperative courses were uneventful. Follow-up examinations under anesthesia allowed for laser augmentation in the first case; no additional avascular retina was noted in the second case, and no further laser treatment was applied. At the most recent follow-up, both babies exhibited well-attached retinas with regressing ROP. Refraction was performed, glasses were prescribed, and close monitoring was advised.

BLRD in zone 1 ROP presents significant therapeutic challenges. Successful management relies on prompt and coordinated teamwork among ophthalmologists, neonatologists, anesthetists, and opticians. Optimal outcomes depend on maintaining adequate hemoglobin levels, timely surgical management, examinations under anesthesia (EUA), laser augmentation, visual rehabilitation, consistent follow-up, strong parental involvement, and ongoing multidisciplinary collaboration.

## Introduction

A retinal vascular condition known as retinopathy of prematurity (ROP) is frequently observed in premature newborns and is the leading cause of infant blindness in both developed and developing nations [1]. Despite confluent laser treatment, aggressive retinopathy of prematurity (A-ROP) and zone 1 ROP are less responsive to laser therapy, and the condition may progress [1]. The world has increasingly adopted primary anti-vascular endothelial growth factor (VEGF) treatment for A-ROP, resulting in better anatomical and functional outcomes [1,2]. Surgical intervention is typically necessary for advanced stages of ROP, specifically stages 4 and 5 [1,2]. A-ROP is a serious and progressive form of ROP that does not follow the classical progression from stage 1 to stage 5 [2]. It advances by rapidly forming fibrous tissue that extends from the optic disc towards the posterior lens surface [2].

According to published literature from Asia, about 3.12% of all premature babies develop A-ROP, which requires treatment in all cases [2,3]. Despite receiving intravitreal anti-VEGF treatment with or without laser photocoagulation, it has been reported that roughly 22% of patients with A-ROP eventually develop tractional retinal detachment (TRD), which is higher than that observed in type 1 ROP [3]. Additionally, two subsets of A-ROP have been identified: posteriorly located TRD, which has recently been described as "volcano" detachment [4], and a combination of exudative and tractional detachment, also known as "bleb" or "blister" ROP [4,5,6]. Both subsets require a rapid sequence of multiple treatment modalities to achieve favorable outcomes [5,6].

## Case presentation

Case 1

**Figure 1:**
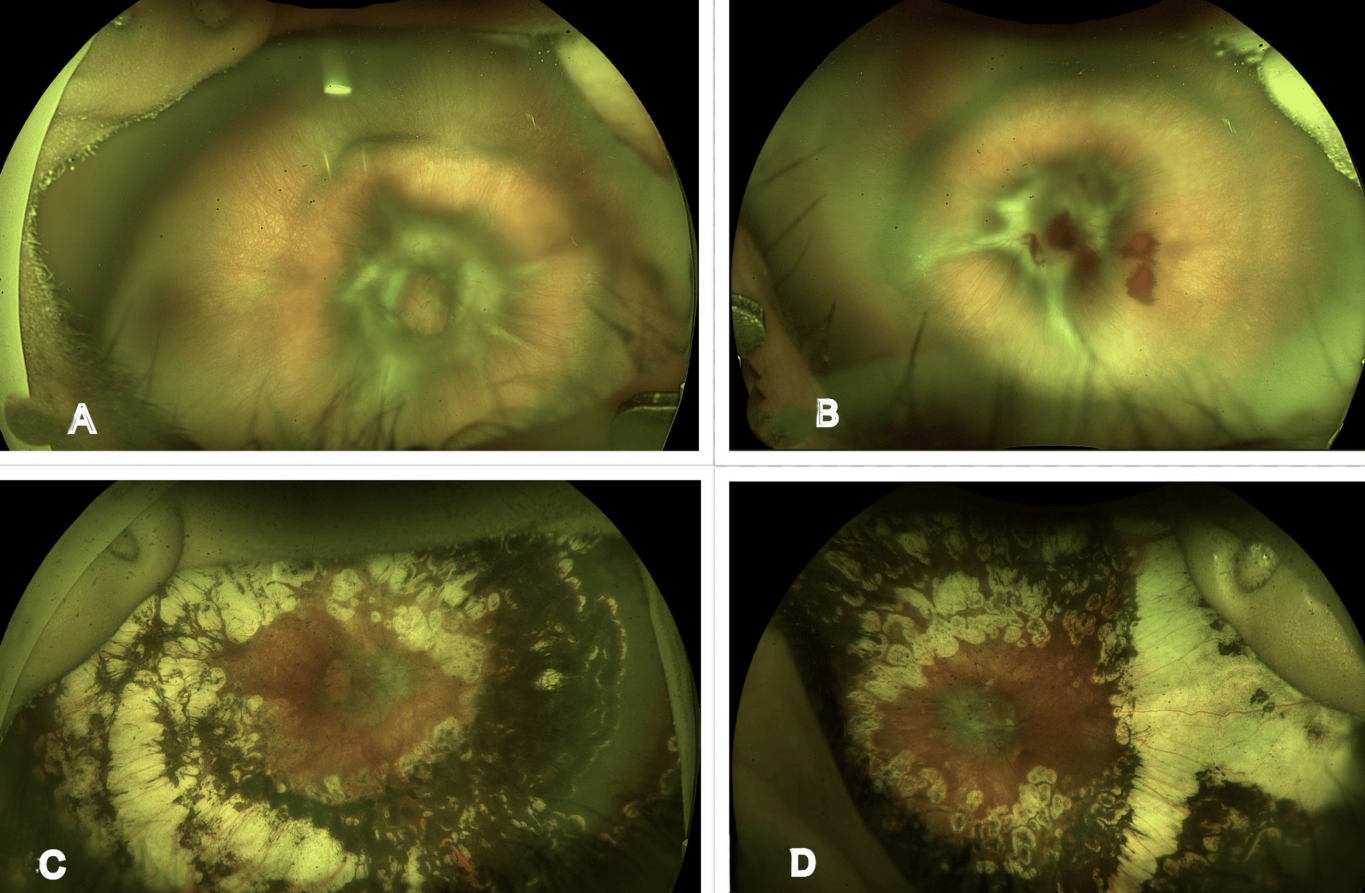
Case 1 images (A, B) A preoperative ultra-widefield fundus Optos image of the right and left eye, showing A-ROP in zone 1 with bleb-like posterior combined retinal detachment, optic disc being obscured with a tractional component, and anterior avascular retina. (C, D) Postoperative ultra-widefield fundus Optos image of the right and left eye at the last follow-up, showing well-attached retina, with optic disc partially obscured and well-lasered retina A-ROP: aggressive retinopathy of prematurity

A preterm male infant born at 28 weeks of gestation with a birth weight of 1600 grams was referred to our clinic for ROP evaluation. The baby’s postmenstrual age at this visit was 37 weeks, with a current weight of 2,500 grams. He had a 15-day neonatal ICU (NICU) stay for respiratory distress syndrome and required oxygen supplementation. The baby was diagnosed with A-ROP in both eyes and had received intravitreal Ranibizumab 12 days earlier at another facility. He was referred to our center for further examination, and an ultra-widefield fundus photo taken with Optos revealed bleb-like posterior combined retinal detachment in both eyes, involving the posterior pole with peripheral avascular retina, and pre-retinal hemorrhage in the left eye (Figures 1A, 1B).

Given the urgency of the situation, the parents were counseled regarding the need for surgical intervention, and consent was obtained. The baby was scheduled for lens-sparing vitrectomy with endolaser and fluid-air exchange. However, blood hemoglobin revealed anemia (Hb: 8.6 g/dL), and hence the baby underwent a blood transfusion, and the surgery was postponed. Meanwhile, the baby received laser treatment using an indirect ophthalmoscope under topical anesthesia on the same day. Surgery was subsequently rescheduled two days later, after obtaining surgical clearance from the neonatologist following correction of anemia.

On the first postoperative day, the retina was well attached under air, with peripheral laser scars visible in both eyes. At a subsequent postoperative visit three months later, the baby was examined under anesthesia. Traction at the posterior pole was observed, with the optic disc partially visible, and laser photocoagulation was augmented posteriorly and anteriorly in both eyes using indirect ophthalmoscopy. The persistent anterior and posterior avascular retina was treated with laser under anesthesia using laser indirect ophthalmoscopy (LIO). This intervention resulted in gradual further regression of the posterior fibrovascular proliferation, which continued to obscure the disc at subsequent follow-up.

There was a need for repeat examination under anesthesia (EUA) after successful surgery due to ocular growth and the occurrence of laser skips in the retina. Following these active interventions, the child was regularly monitored. At the last follow-up, the child had reached one year of age, with both eyes showing complete retinal attachment, peripheral laser scars, and partial obscuration of the optic disc by membranes (Figures 1C, 1D). Refraction revealed -2.75DS/-1.50DC ×180 in the right eye and -2.00DS/-3.00DC ×170 in the left eye. TAC vision in both eyes was 20/320. The child was prescribed glasses and encouraged to engage in outdoor activities, with frequent follow-ups recommended for close monitoring.

Case 2

**Figure 2:**
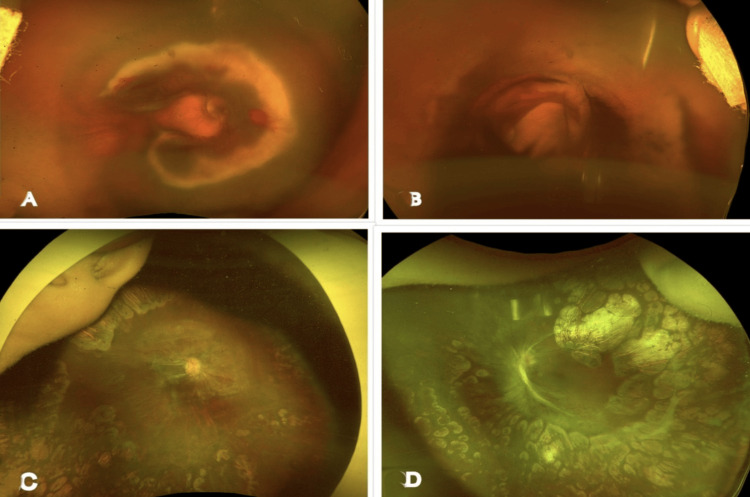
Case 2 images (A, B) A preoperative ultra-widefield fundus Optos image of the right and left eye, showing A-ROP in zone 1 with bleb-like combined posterior retinal detachment, optic disc being obscured with a tractional component, and an anterior avascular retina. (C, D) Postoperative ultra-widefield fundus Optos image of the right and left eye at the last follow-up, showing well-attached retina, with optic disc partially obscured and well-lasered retina A-ROP: aggressive retinopathy of prematurity

A preterm female infant born at 28 weeks of gestation with a birth weight of 650 grams, previously diagnosed with A-ROP in both eyes and having received an intravitreal ranibizumab injection 10 days earlier at another center, was referred to our hospital for ROP management. The baby’s postmenstrual age at this visit was 34 weeks, with a current weight of approximately 1200 grams. She had an eventful postnatal course, including a 42-day NICU stay and 20 days of oxygen supplementation. The baby had also received a blood transfusion for the correction of anemia. An ultra-widefield fundus photograph taken with Optos revealed stage 4B zone 1 disease in both eyes, with bleb-like combined posterior retinal detachment involving the posterior pole and peripheral avascular retina (Figures 2A, 2B).

The baby was scheduled for lens-sparing vitrectomy with endolaser and fluid-air exchange in both eyes; however, due to a delay in obtaining surgical clearance, the baby underwent laser treatment using an indirect ophthalmoscope under topical anesthesia on the same day. Two days later, the planned surgical intervention was performed. On the first postoperative day, the retina was well attached under air, with peripheral laser scars visible in both eyes. The baby was regularly followed up, and regressing ROP was observed at each visit. Examination under anesthesia showed no additional avascular retina, and therefore, laser augmentation was not performed. At the last follow-up, when the child reached one year of age, ROP had regressed, with a well-attached and adequately lasered retina (Figures 2C, 2D). Refraction revealed +1.00DS/-2.50DC ×180 in the right eye and +0.75DS/-2.50 ×180 in the left eye. TAC vision in both eyes was 20/320.

## Discussion

This report describes two premature infants born at 28 weeks who presented with A-ROP in both eyes within zone 1, complicated by combined bleb-like retinal detachment (BLRD). The cases highlight the importance of early detection and timely intervention, particularly in posterior zone 1 ROP, where uncommon features such as BLRD can lead to severe vision loss. The International Classification of Retinopathy of Prematurity (ICROP) 2021 has not yet included this variant, although it is gaining attention due to its complex treatment requirements and poor visual outcomes [4,5]. Some atypical forms of A-ROP reported in the literature include exudative retinal detachment, giant retinal tears causing closed-funnel RD after laser, severe fibrovascular proliferation on the optic disc, and hybrid ROP, characterized by the presence of both ridge tissue resembling staged ROP and areas of flat neovascularization mimicking aggressive ROP [7,8,9,10].

The cases we report here are atypical in that both infants developed bleb-like combined posterior retinal detachment. Since VEGF drives ROP, anemia may play a significant role in this complex disease. Correcting anemia can improve oxygen delivery and reduce VEGF levels in the eye [11]. Both infants in our report had a history of blood transfusion, illustrating the potential contribution of anemia to BLRD. The exact pathogenesis of BLRD in ROP remains unclear. In severely ill neonates with ROP exposed to 100% unblended, unmonitored oxygen, bleb-like posterior combined exudative and tractional retinal detachment may develop, likely reflecting a form of oxygen-induced retinopathy. The bleb-like configuration of these detachments may result from exudation from immature blood vessels, worsened by shearing forces from contracting fibrovascular tissue, and limited to the posterior disc and immature macular tissue [5].

Although retinal detachment can result from both anti-VEGF crunch and BLRD, these conditions differ in their underlying mechanisms and clinical appearance. An atypically shaped, bleb-like retinal detachment is predominantly exudative with a combined tractional component, whereas anti-VEGF crunch is associated with the formation of fibrovascular tissue and increased traction [5,12]. Management of BLRD can be highly challenging, often requiring multiple strategic approaches. These may include intravitreal anti-VEGF injections, laser photocoagulation, and, ultimately, vitrectomy. Timely surgical intervention has shown promising outcomes. The unique configuration of BLRD, the location of poorly differentiated retinal tissue, and disorganized vasculature make vitrectomy particularly difficult.

In our cases, we demonstrated that prompt intervention, including laser and surgery, can lead to favorable anatomical and visual outcomes. Another important aspect addressed was systemic management, including referral to a neonatologist and timely blood transfusions. The pathogenic significance of low hemoglobin in ROP development may be explained by tissue hypoxia and increased VEGF release caused by reduced hemoglobin levels in preterm infants with developing retinas. Maintaining higher hemoglobin levels in preterm newborns may improve hemodynamic stability and reduce complications associated with prematurity [13]. This report also emphasizes the importance of examination under anesthesia during follow-ups and timely laser augmentation to halt disease progression, highlighting that ROP management is not a one-time intervention.

## Conclusions

This report describes two cases of BLRD in infants with A-ROP. Both of the cases were successfully managed. Key factors contributing to successful outcomes included intravitreal anti-VEGF, LIO, timely lens-sparing vitrectomy surgery, maintaining adequate hemoglobin levels, frequent EUA, laser augmentation, refractive correction, and strong parental involvement. Postoperative recovery was smooth in both cases, with well-attached retinas and regressing ROP observed on follow-up. The report emphasizes the importance of multidisciplinary teamwork in managing complex ROP cases with BLRD. Effective management is achieved through prompt collaboration among ophthalmologists, neonatologists, anesthetists, and opticians.
